# The Inanimate Third: Going Beyond Psychodynamic Approaches for Remote Psychotherapy during the COVID‐19 Pandemic

**DOI:** 10.1111/bjp.12720

**Published:** 2022-03-01

**Authors:** Sari Goldstein Ferber, Aron Weller

**Keywords:** REMOTE PSYCHOTHERAPY, COVID‐19, PSYCHODYNAMIC PSYCHOTHERAPY, HUMANISTIC APPROACHES, LONELINESS, HOPE, INTERSUBJECTIVE PSYCHOANALYSIS

## Abstract

The COVID‐19 pandemic exposed the field of psychotherapy to the need to provide treatment remotely. We discuss the question of whether remote therapy can be curative and if the electronic device used to manage these sessions unites or separates the therapist and the patient. We term the electronic device as ‘the inanimate third’ in the therapeutic process and discuss the objectivity of the device as opposed to the subjective emotional processes involved. We deal with emotional themes relevant to the COVID‐19 pandemic and associated social distancing practices, such as longing, loneliness, the perception of the future and the lost past, and the efficacy of the therapeutic stimulation of fantasy and hope. We also evaluate the possibility of existing transference and countertransference processes while working remotely. We suggest the term ‘social paradox’ to describe the situation in which an objective entity such as the digital media symbolizes both distance and intimacy as well as separation and unity. We conclude by stating that containment of the social paradox by the therapeutic dialogue is possible as the existence of the dialogue eliminates elements of the paradox.

## INTRODUCTION

The COVID‐19 pandemic exposed societies worldwide to increased virtual work with cell phones and computers. This last year has been termed as ‘the year of sad passions’ (Facchin, 2021), placing COVID‐19 pandemic survivors in danger of symbiosis and omnipotence while confined at home, dependent on videoconferencing. This year has also been characterized as the next drive for psychoanalysis to transform and modernize towards greater efficacy and maintenance of hope (Facchin, 2021).

Psychodynamic approaches adopted the use of videoconferencing from their early utilization for public use (Dettbarn, 2018; Russell, 2015, 2021). While detailed descriptions have been published on the distortion of the analytic encounter by technical features of the electronic devices, their hitches and mishaps (Brahnam, 2017), others have recently shown the validity of the analytic encounter using electronic devices (Lemma, 2017). Specifically, in order to perform effective treatment online one needs to maintain a sense of presence. This is a neuropsychological state, which occurs for all living species. Online presence is a state termed ‘telepresence’ (Russell, 2015, 2021). Telepresence is an experience of the state of presence where awareness of the mediating device temporarily recedes or disappears.

When considering the mental experience of reality, Deleuze (Biehl & Locke, 2010) claims that the virtual reality is real although opposed to the actual reality. Deleuze uses the word ‘becoming’ to describe the continuation of virtual events. This process of experiencing real emotional progression within a virtual reality has been explained by others who coined the term ‘embodiment’, meaning bodily sensation and encoding of any social sensory input. According to the Boston Change Process Study Group (The Boston Change Process Study Group, 2013, 2018) our bodily sensations encode any reality, being it a true or a virtual social encounter. The discovery of mirror neurons, which enable us to perceive our ‘self’ as well as the other's in the same event, makes it possible to understand that these neurons work to establish a perception of the other seen on the screen. This is suggested to be a perceptual process, which in our view coincides with the concepts of reverie, reflection and emotional meeting between the patient and the therapist within their shared fantasies and experience of the analytic third (Benjamin, 2017).

We aim to show that although the sensory input is altered when using virtual reality, the output, the embodiment, which continues the analytic process within the meeting between the therapist's and the patient's fantasies unfolding unconscious to conscious, still exists. Embodiment of the patient and therapist may turn the electronic device, the inanimate third, alive, somewhere within the mutual fantasies of the parties in the analytic encounter. In this sense, we follow the intersubjective school in psychoanalysis, emphasizing subjectivity and intersubjectivity in contrast to the search of an absolute truth, which indeed may be distorted by technology. However, is there an absolute truth in face‐to‐face meetings and is that truth the true goal of the analytic process? The requirements for a virtual encounter remain the same as those needed for face‐to‐face and couch meetings: they basically point to the elementary prerequisite of the patient's and therapist's levels of availability for reverie which is a very complex process of perception driven analysis and goes beyond the identification of the sensory input source for its utilization and efficacy.

This paper aims to present manners of virtual interaction during the ‘digital age’ (Lemma, 2017) by raising the question of whether use of the electronic device termed the ‘inanimate third’ can serve the psychotherapeutic aims and the patient's needs, and if this may open new horizons for psychoanalytical treatment. The term ‘inanimate third’ suggests both a symbol of separation between therapist and the patient, and at the same time a symbol of unity and social overcoming in the social distancing era and in conditions of geographical distance. It is inanimate, but it facilitates social engagement in conditions of forced social restrictions. Thus, we find that the electronic device is projected with an unspoken double truth symbolization: Separation, which could induce the rise of past social losses and conversely a sense of unity, which could arouse the possibility for reparation of a loss. It is the third participant, although inanimate, between the two engaged humans, the patient and the therapist. We note that if both parties have extensive experience with digital media communication, its role as a ‘third’ may be diminished by some extent of habituation to its existence.

An important question is whether remote psychotherapy can still promise curative aspects for the patient. Are the social distancing restrictions opening our eyes to the post‐modernistic future and the next generation of human contact or whether they represent just a reduction of traditional well‐accepted face‐to‐face methods of psychotherapy? Does remote psychotherapy represent diminution of the psychotherapeutic encounter or rather breaking through to new international borderless horizons?

## THEMES FOR CLINICAL REMOTE APPLICATION DURING THE SOCIAL DISTANCING ERA

### On Broadening the Therapeutic Communication

The psychotherapeutic encounter has been differentiated from plain social encounters ever since its inception. Even periods of silence during psychotherapy were recognized as essentially different (Cook, 1964; Ferber, 2004; Levitt, 2001). During the pandemic, virtual psychotherapy is a source of social support, with the risks that it will become a simple non‐curative interaction. Communication via the ‘inanimate third’ is narrowed by the fact that there is no direct eye contact, just an illusion when both patient and therapist are looking at the camera. In fact, all the therapist knows is whether the patient is looking into the camera or not. The therapist, as much as the patient, may be distracted by digesting the patient's manifest and latent contents and take his/her eyes off the camera and screen. A different distraction is that both the therapist and the patient view themselves on the screen in addition to viewing the other. In the face‐to‐face interaction we are not perceiving ourselves visually. We perceive ourselves mentally. This distraction may overwhelm both the therapist and the patient and take the therapeutic encounter to the field of performance anxieties. Note that in most platforms, however, the ‘self‐view’ can be hidden, although performance anxiety may still arise in front of the camera.

To overcome the limitations of work with the ‘inanimate third’ during the social distancing era, the major aim is to reach a gradual developing alliance with the analytic process from confused levels at the beginning of the session to a more elucidated, clarified, organized and contained state at its end. Following a few therapeutic sessions, the patient may instinctively internalize the analytic process and reach a more self‐contained and emotion‐regulated condition. The significance of the therapist−patient co‐regulation process with regard to the patient's analytic aims may broaden the communication from simple content analysis to complex parameters of sequencing and dynamics of emotional processes.

The COVID‐19 pandemic has serious implications for mental health. It involves stress exposure and stress reactivity (Unützer, Kimmel & Snowden, 2020). Arousal states are part of the human daily affective reactivity, within the adaptive range between susceptibility and resilience to stressors. The individual's resilience is measurable by the effectiveness of ending those arousal states, among other aspects of resilience (Ferber *et al*., 2021). The challenging issue is the ending of the arousal state while the stressful situation continues (coping with chronic stress). For many, the reduction of arousal and development of healthy coping mechanisms occurs in levels beneath self‐awareness and consciousness. The prolonged pandemic situation and its mental health consequences may challenge many with the need for conscious affect regulation and arousal modulation (Bradley, 2003). Virtual therapy could address this issue in the current tough times.

As early as 1927 (Freud, 1955), Freud related to World War I as an external worldwide stressor, which according to his postulates generated mental pathologies. He emphasized the element of surprise as the source of ‘traumatic neurosis’: ‘fright (Schreck) is the name of the condition to which one is reduced if one encounters a danger without being prepared for it; it lays stress on the element of surprise’ (1955, pp. 12 − 13). He also suggested that internal stressors are perceived as external stressors ‘in order for it to be possible to apply against them the defensive measures of the barrier against stimuli (Reizschutz)’ (pp. 12 − 13), laying the basis for the understanding of projection.

Thus, when external worldwide stressors such as the pandemic meet the patient's internal stressors, the working through lies beyond predominant interpretation and stays within the subjective therapist−patient interactive dynamics. This ranges from the outburst of stressful reactions phenomenology at the beginning of the session to a desired positive transference state recognized by both the patient and the therapist, including positive affectivity and its aligned verbal and non‐verbal manifestations. The dynamics of this process have been defined earlier as ‘co‐regulation’, suggesting that mounting arousal states are worked through together until reaching a return to a balanced state (Ferber, 2008). These co‐regulatory aspects in the communication between the patient and the therapist (Stern, 1985) during the existence of the dysregulating pandemic factors are essential for the remote communication and may help broaden the communication.

The end of the session with a quiet positive transference may be compared to a child's needs for a story to terminate with a ‘happy end’ in order to support his/her thriving to grow and develop. The mature mind similarly needs to experience coherence and positive affect for the development of coping and resilient strategies within an objectively insecure situation during the pandemic. As the human brain functions to avoid extreme excitatory and extreme inhibitory conditions at any given point (Goldstein Ferber *et al*., 2021b) as much as possible, this may support a subjective sense of security and adaptive reactivity to the pandemic‐related stressors.

*Vignette 1* ^1^: A couple, Mr A and Mrs A, attending parental guidance sessions, shared their stressful experience in the quarantine situation and complained that the condition of their son is worsening. They described a fearful scene in which the child aged 12 took a sharp knife and threatened his father that he intends to kill him. The second fearful scene they described was about the child holding matches in his hands and threatening to set the house on fire. The parents added that the two months of quarantine are too much for them and for the child. The parents also shared their concerns about the child's internet gaming addiction which has been worsening since isolation onset. I said that I share their concerns and that probably the feeling is of being jailed and that the child is unconsciously presenting his rage by trying to provide a legal cause for being jailed as the coronavirus is a less comprehensible cause. Both Mr A and Mrs A were tearful and expressed their helplessness and their love for the child. They also expressed their fear and I asked if the fear is a part of their daily affairs with the child during the quarantine. I asked to see the child and after a long time he agreed to come to the room with the camera but stayed off the camera. I said I appreciate his willingness to talk to me. I said I understand that sometimes fears create fearful activities. The child asked who is fearful except him. I answered that his parents and me are fearful. The child was surprised. I asked then how he is feeling while talking to me. In response he came to the front of the camera. I said I appreciate that he struggles to show himself to me and asked if he is willing to describe some of his fears in a drawing. It took some time for him to answer and I continued to appraise his talents. He then asked if I request the drawing now. I said: ‘pick a comfortable place in your house, draw a picture of your fears and see if you want to come back to the camera’. The parents reacted with relief to the fact that the child cooperated with me. This was the first phase of eliciting positive transference by the freedom to choose. After 10 minutes outside the room the child forced his way into the room and demanded to show his picture. When viewing the picture, I said: ‘but the family members in your picture are so small’. The child reacted by running out of the room. I asked his father to explain that I am not used to partial communication and I ask him to please come back to the camera. The child came and sat down off the camera and said: ‘You criticized my picture’. I answered that I am trying to understand him and why the figures in the picture are so small. I added: ‘do you feel that all of us are so small?’ The child came back to the camera and said: ‘we are all small. You too. We do what we are told to do, and we are told to be isolated without schools and friends’. I asked about his stress. He answered that he understands his drawing better. ‘I fear that I am smaller than my age and my parents too. We all obey rules. You too’. I acknowledged his fear and asked about his fantasy to destroy rules. I glanced at my watch and wondered if we have enough time to consolidate the interaction cycles and return to balance until the end of the session. The child said there is no need to acknowledge his fear because we are getting smaller and smaller and this is the end of the world. He said: ‘We are jailed in the house and soon we will not be at all’. I said: ‘so the small figures in your drawing is a close up of a process of losing the world?’ The child said: ‘well not really… I am sorry … you are nice … when can I see you again? I will go to sleep now’.

*Interpretation*: This was the end of a long communication cycle composed of many mini communication cycles which was ended by the child's age‐appropriate wish to go to sleep quietly. The manifest content regarding the pandemic and the quarantine situation had a latent dynamic content of dealing with the child's unresolved Rapprochement struggle (Mahler, Pine & Bergman, 1975), including the acceptance of non‐omnipotent reality which underpinned his healthy separation‐individuation (Mahler, Pine & Bergman, 1975) component in his wish to end the communication and go to sleep. This developmental supportive acceptance was temporarily achieved by the child at the end of the session, showing a stable enough condition and a return to balance when presenting his wish. The repetitive cycles of communication, through successively touching the trajectories of de‐omnipotence, used the verbalization of the external stressor concomitantly with the underlying dynamics of handling a Rapprochement crisis, providing benefits for the given pandemic situation as well as for the child's continuous internal unstable affectivity. Furthermore, these repetitive communication mini‐cycles were oriented at, and successfully built up, the child's ‘apprehensive preparation’ (Freud, 1955), a condition which is absent in affective reactions to a threat perceived by surprise, and which according to Freud, comprises the first line of defence against dangerous stimuli (Freud, 1955) such as the pandemic. To conclude, this session was about emotional survival of a child threatened from within and from the outside situation. He talked about physical survival of the human kind but that represented his fears to emotionally collapse undefended. The ‘inanimate third’, the computer that he so much loved to play with, served as his defence on that night.

### Mutual Perception of Self and Other

The perception of self and others is a key aspect of social cognition. Our ability to explain and predict other people's behaviour by attributing to them independent mental states, such as beliefs and desires, is known as a ‘theory of mind’. The perception of the other is based on a developing cognition which matures around the age of 4 years, a subjective belief by which the ‘other’ resembles the ‘me’ (Doherty, 2009). Functional imaging has played a key role in seeking to identify brain regions specific to this ability (Gallagher & Frith, 2003).

Meeting between minds is dependent on the perception of self and others in the individuals participating in the interaction, but this is not exclusively related to face‐to‐face interactions. This may be extended to remote psychotherapy. The existence of the ‘other’, the therapist, even when perceived via the ‘inanimate third’ contains many curative aspects.

Mental curing was historically related to ‘conflict resolution’ (Freud, 1942). This term was originally related to internal curative processes of the patient. More recent progress in psychotherapy gradually related such curative aspects to the therapist−patient interaction and claimed that the cure results from the interaction within this dyad (Aron, 2006; Baker & Baker, 1987; Cashdan, 1988; Kernberg, 1967; Kohut, 1977; Mitchell *et al*., 1999; Ogden, 2009). However, the therapist's role remained dominant in these therapeutic relations, although as a subjective and not as an objective entity as originally this role was thought to be. From a humanistic perspective, during the pandemic, the therapist maintains the professional attitude and the setting of the therapy sessions, even while conducting an eye level dialogue.

The therapeutic session using the ‘inanimate third’ stays curative by means of enhancement of coping strategies and individual strengths of the patient. The human capability of mutual perception does not have to rely necessarily on real face‐to‐face interaction. The mutual experience of struggle during the session and the efforts of the therapist to reduce the patient's levels of disengagement may account for the virtual walk taken together during the session. Sharing a mutual perception of self and other with the therapist may empower the patient during these tough times.

It is also suggested here that the therapeutic process occurs in numerous interaction cycles started and ended by either the therapist or the patient (Stern, 2002) and that the dialogue which is composed of all the interaction cycles in the session has curative potential. The content of the conversation may not always be of utmost importance. Instead, the progression of the dialogue, the creation of mutual perceptions of the self and other, and its aligned emotion regulation have important curative roles. These moments of togetherness during the session provide the opportunity for reaching a mutual perception. The moments of sharing a mutual perception may be limited but precious and curative.

A further curative aspect may be apparent in the accuracy of therapeutic perceptions. This may be achieved by using the patient's verbal content, both manifest and latent, for the expression of the therapist's mentalization of the patient during the session, thus fulfilling the deepest wish of any patient, which is to be understood. The patient's positive feeling of being understood may be achieved by the therapist's efforts to create with him/her a mutual perception. In these difficult times of the pandemic and the related national and international chaos, this feeling of being understood may turn out to be like a flickering of light in the darkness.

*Vignette 2*: A young lady, Ms B, aged 17, began the session with sharing her experience of participation in a demonstration aimed at protesting against men's violence towards women. She shared her positive experience in the demonstration and mentioned that it was good despite the requirement for social distancing and masks. While acknowledging her positive experience I mentioned that we discussed in the past our mutual feminist views and the writings of Simone de Beauvoir that she keeps reading and I read them when I was her age. She said in response that it feels good to share the same ideas and life concepts: ‘The fact that you are willing to identify with my extremely young ideas feels that you are together with me in these ventures’. I replied that our minds crossed as I had the same feeling and that we cross the screen. Thereafter she mentioned the high toll of women sacrificed by their husbands during quarantine. I mentioned that one of my colleagues was appointed to diagnose the man who is suspected to be the murderer of his girlfriend last week. She answered: ‘I heard about it on the news. It was mentioned that there is no clear diagnosis because the subject tried to cheat the evaluators’. Then a long silence occurred and I wondered if I lost her and that it's so easy to turn off an electronic device. I asked: ‘Are we frightened by men?’ She answered that she was thinking about her father and her boyfriend. I said: ‘so you realize that there is still love in the world and that feminism that brought us together at eye level is not an ultimate philosophy without limitations’. She said many thanks for bringing up the concept of love. She then elaborated on how she is worried about her boyfriend's wellbeing who told her he feels bad while serving in the army. She needed support at the moment she recognized her caring attitude towards the men in her own life. Following my support, she said: ‘my boyfriend and I are musicians. This just has to do with love for life and people’. I commented: ‘This is your own voice, your own statement, regardless of books, public events and educators’.

*Interpretation*: This shows that the mutuality in the therapeutic encounter while working remotely during the pandemic does not reduce professional status nor the option to cure and bring the patient to realize her own individual needs and concerns. On the contrary, the mutuality may empower the patient and support her courage to touch difficult internal conflicts within her own perception of self and other instead of simple child‐like imitations of global ideas as is.

### On the Therapeutic Permission to Feel and the Patient's Subjective Feeling of Freedom

Any therapeutic encounter may need to focus on how the patient feels. This identifies the goals of the meeting and opens the arena of helpful therapeutic attitudes. These issues focus on the patient's needs and keep the encounter curative as well as maintaining the therapist's role. The patient may lack the required permission to experience emotion, which is essential for emotional stability during social restrictions and the quarantine situation may raise many aspects of feeling emotionally imprisoned. Beyond verbal assurance and acceptance, the curative therapeutic talking may need to provide the patient with the permission to feel by acknowledging and recognizing the underlying emotions during the talking. The patient may not have this internal permission without therapeutic guidance and may pay high emotional costs as a result. This therapeutic permission conveyed by the therapist has the potential to contribute to the patient's perceived mental freedom. In situations of controlled togetherness such as social distancing, the permission to feel and perceived freedom are highly valuable.

The subjective lack of feeling freedom resulting from the social distancing restrictions is more prominent than the objective restrictions of remote therapy. Remote psychotherapy entails both curative entities and aspects of broadened communication by providing the opportunity for therapeutic working through of the issue of freedom, including the acknowledgement and support of the legitimated emotional freedom of the patient. That is, the identification and acknowledgement of subjective actual emotions may result in perceived emotional freedom while being verbally contained by the therapist. This acknowledgement of perceived emotional freedom also maintains the therapeutic encounter via technology as a true therapeutic encounter in the era of the pandemic.

The transfer from concrete to abstract cognition in brain functioning has been recently discussed (Gilead, Trope & Liberman, 2020). As feeling better is what counts in psychotherapy, awareness to one's subjectivity may be felt as crossing the borders of the screen. It is also near the concept of transforming the present worldwide situation of controlled freedom to the personal narrative of patients’ wishes and needs for emotional freedom.

The permission to feel through the ‘inanimate third’ may be transformed in the patient's mind as the permission to live both mentally and physically, a transformation of utmost importance during the pandemic. It puts the therapist in a position of exerting efforts to help and support. This perhaps represents the most intimate moments between the therapist and the patient, showing both containment and holding of the patient's situation by bridging the gap of distance.

*Vignette 3*: A young lady, Ms C, aged 16, was emotionally blocked in the beginning of the session. Then she said she is under a lot of stress with the return to school given social distancing regulations that cause the teacher to confuse the students. She trembled and told me she is sweating and that she can't speak and can't think. I acknowledged her inhibitions. I asked if it is difficult for her to speak out because she feels angry. She said that she does not like herself when she is angry. I said she has some self‐esteem concerns when angry. She said: ‘yes, first we were quarantined and worked on the Zoom which was very hard for me and now nothing is really back to normal’. I answered that she begins to express her rage towards the educational system and the era of social distancing. She continued to express her frustration by being forced to meet with friends with masks and distance and no touch. I acknowledged her quest for freedom and independence, which is common at her age. She answered that the anger and rage are blocking her from dealing well with homework and exams. Then she had an idea to make a written plan of the next hours. I said this may enable her to feel more control over her life. She left the camera and after a few minutes came back with her notebook and for a long time we kept silent while she was writing up her plan. I wondered during the long silence if the screen will still be there for her after she completes her plan. My concerns evaporated when she turned to the camera and said that without being able to express her rage she would not have found any control on her life. I answered that her freedom and independence will be apparent by the control she will have during the next coming hours. She said she desperately needed it as in a few hours she has to take an exam.

*Interpretation*: The legitimation to feel and express her feelings paved the way for Ms C to the feeling of freedom in the face of accumulating demands, which in turn worked out well for her to feel some control over her life. Freedom is not given, it is taken when emotionally recognized.

### On Loneliness

Researchers in informatics suggest that the state of telepresence is never consistent online and it alternates with an awareness of being truly alone in the absence of the other (Russell, 2021; Turkle, 2011). The predominant impact of the social distancing restrictions is loneliness. Earlier views of loneliness connected this experience to risks of developing psychopathology (Erzen & Çikrikci, 2018). However, positive views of aloneness have been noted in the past and they have been related to improved development of the child if a caregiver is silently present in the child's physical environment. This has been formulated in the notion of ‘being alone in the presence of someone’ (Winnicott, 1958). The rescue from being alone during the pandemic may be attained, in addition to phone calls and digital social networks, by communication between the patient and his or her psychotherapist through the ‘inanimate third’. The cell phone or the computer may provide a transcendent space where a meeting between minds occurs. This has been addressed in the earlier view of psychotherapy as ‘being instead of doing’ (Green, 2010).

The fact that the psychotherapist is available on the screen may be a different type of loneliness as it is experienced while the therapist is available. This availability may stimulate self‐awareness which has been noted earlier as supporting mental health (Parloff, Kelman & Frank, 1954). This of course is not the original meaning of ‘being alone in the presence of someone’ but may be translated to working with the ‘inanimate third’ by proposing that higher order perceptual processes are available when being in social contact while being physically alone.

The curative perspective of working with the ‘inanimate third’ lies in the patient's subjective assumptions on his/her role in the therapist's mind, which in turn may buffer feelings of loneliness and detachment. Being valuable to the recognized ‘other’ has been noted as missing in various mental disorders (Mitchell *et al*., 1999). Recognition and acknowledgment by the therapist has been regarded as curative along the history of psychotherapeutic theories (e.g., Reis & Brown, 1999). The remaining question in this regard is whether the remote availability of the psychotherapist is experienced as a reparation of loneliness by partially finding the lost social world.

*Vignette 4*: At the time scheduled to initiate the session, Mr D aged 18, a very reserved and rationally oriented youngster, sent a WhatsApp message that he has troubles with his computers and it will take him a few minutes to fix it. I texted him not to worry, I will give him extra time as we are both dependent on technology and need to be flexible. When he logged on, he started to share his lonely week. He said that many of his social contacts avoided texting and telephone calls and responded after a long time if at all. He added that his best friend recovered from his low mood condition and has a new girlfriend, so his time for Mr D became limited. I asked if he is anxious more than last week and asked if he wondered if he will have enough time and enough support using technology. He said it's different and that he prefers face‐to‐face meetings especially when being so lonely this week. I asked him if he wonders whether I can treat loneliness when being afar. He said logically it does not sound feasible. I asked if I am a replacement for his lost social world or a facilitator to enhance his social engagements. He said that he is aware that I am not among his social circles but he had a bad time at home with shouts between his mother and his sister, and in a way his rational father has been his rescue. He added that he is able to be calm, but he is looking for happiness in his life in addition. I asked if he found happiness in his family in the past. He answered: ‘Never, the best situation I could expect is a quiet and calm atmosphere’. I mentioned that children are usually happy when playing and asked about happy moments in his childhood. He described in reply a few scenes with a friend when he was 10 years old and both of them were running to the friend's grandmother's house and her large video room. I mentioned that he connects happiness to a close relationship within his peer group and to enjoyment of video. He said: ‘Actually, I have a good feeling about that video’. I asked: ‘and how do you feel now?’ He answered that I take him to good perception of his peer group and that the current discussion is very good. I said: ‘What was so different this week that left you emotionally low and reserved like your family and avoiding the others that you enjoy?’ He complained about paranoid‐like thoughts on his friends. I said that becoming crazy in this new era of the pandemic is easy and asked him in which part of reality he would like to place himself. With his mother and sister, with his rational father or in another way he would invent for himself and his social contacts? He said he can't complain about his friends as he was too busy this week and for some reasons missed some social encounters. I said that I recognize all his good will and social capacities. He asked in reply: ‘Do you really?’ I replied that: ‘I understand you feel lonely as we have been informed that we are back to normal and that only the social distancing remains as a regulation. Maybe we are lonely because we are not going back to the lost social world but going forward to a new reality?’ I mentioned that we have many uncertainties in this new reality and that he had many uncertainties as a child when his parents avoided moments of happiness, and that he probably wondered about it. I also asked him if he prefers a bad paranoid reality in order to overcome the anxiety related to uncertainty. He said that this is beyond his logic, but it touches his heart. I shared my excitement about these words. He dried up his tears and said that he usually feels such feelings when meditating. I said that this is a hug he gives himself and he answered that he wonders if this is truly a hug. I said: ‘You are alone in your meditations but not lonely, and don't feel lonely now as you are emotional while being alone in my (remote) presence’. He stated that his worries and paranoia‐like thoughts were reduced. I said that generally speaking, when you are lonely in the presence of someone you are not lonely anymore but in contact with your internal emotionality and senses.

*Interpretation*: Being alone in the presence of someone supportive, even remotely, may reduce anxiety and depression posed by the pandemic era. In the absence of social realities, Mr D preferred his negative, threatful interpretations that were identified as partially related to his loneliness during childhood. This attitude was the basis for his paranoid‐like intrusive thoughts while the session provided him with more space for legitimate anxiety during uncertainty. When realizing that he is ‘being alone’ and not lonely he could give up the unnecessary negative feelings and interpretation regarding his existing social circles. The fact that he was able during the remote session to target the uncertain pandemic situation as the cause for his negativity rescued him from feeling falsely secured by a negative paranoid certainty. This corresponds to our recent theorem on uncertainty‐related anxiety during the pandemic (Goldstein Ferber *et al*., 2021a). The memories of happiness supported his more adaptive view on the social distancing era and his chances to become happy as an individual. The ‘good video’ in his childhood supported his elaborated positive view of the ‘bad video’, which is being treated remotely. The attraction of the ‘bad object’ over positive experiences, as anti‐libidinal tendencies, has been mentioned earlier (Sherby, 2007). Additionally, the early theoretical and clinical reference of paranoid‐like thoughts to early failures in sensing gratitude (Klein, 1957; Ogden, 1990) remains to be further worked through with Mr D. This points to current losses due to the pandemic, which probably elicit emotional investment in past losses as an emotional source of continuous grief.

### On the Recognition of Longing and Nearing the Future to Present

Touch is the first of the five human senses to mature in the infant after birth and the last to die (Ferber, 2008). As such, it is extensively challenged by the regulation of social distancing. The worldwide population is not allowed to hug, hold or even shake hands. Although in face‐to‐face conventional therapy psychotherapists do not touch the patient for curative efforts, during the era of social distancing patients may need to be virtually hugged and held. This is occurring on top of the patients’ developing longings for touch in their daily affairs.

The condition of longing may consist of aspects of grief regarding a lost social world, which in turn may give rise to many past memories and issues of loss acceptance. Past theories on reactions to loss ranked those reactions from denial and rage to depressive states (Kübler‐Ross & Kessler, 2005). However, identification with the future of digital media may replace, at least partially, the personal longing and perspective of loss. Looking to the future is always related to the fuelling of motivational instincts, and thus it may replace the sense of a lost world and the tendency of looking to the past. This curative process may buffer the sense of longing and the tendency to avoid which corresponds to the ‘fight or flight’ conceptualization (McCarty, 2016).

*Vignette 5*: Ms E, aged 25, a young lady with many externalizing characteristics and connections within the Bohemian sociality in the big city Tel Aviv, began the session with a statement that she has major problems with her mother who is not compliant with the social distancing regulations. She thereafter detailed that when she visited her a few times the mother hugged her and kissed her. After a few visits Ms E argued with her mother and said she wants to protect her from being contaminated and as Ms E never stopped her social encounters during quarantine she felt she is dangerous and may be a risk for her mother. In her insights she said she recognizes that her mother misses her a lot and wants to touch her. I mentioned the importance of the sense of touch for her mother and the rest of the world, as well as the longing of both the patient and the mother for each other. I said to Ms E that she kept her old social world alive during quarantine and asked if we are looking back to the old social world to maintain past securities. She then asked, what is the future? I answered that she is asking her mother to look to the future of social distancing while she herself may need the continuation of the past. I mentioned that the argument between Ms E and her mother represented the wish of the mother to be part of the surviving social world of her daughter. Ms E replied that she is willing to take a step ahead to the future if she would be able to know where we are heading. A long silence followed while both of us tried to think about answers regarding the future and remained only with existential uncertainties. We further discussed if the future is in our control at all at any time, and that looking to the future is of utmost importance. We also discussed for the first time her unspoken needs for her companion's touch.

*Interpretation*: In this way the longing of both Ms E and her mother that led them to the unnecessary argument has been acknowledged. Ms E was supported in nearing the future to the present beyond her clinging to her Bohemian past. This also triggered her to think about herself and her companion as individuals within the intimate dyad beyond her past as a ‘social butterfly’. This is in accordance with our recent findings that the most useful means for emotional survival during the pandemic are the closest and the most intimate nets (Ferber *et al*., 2022).

### The Stimulation of Fantasy and Hope

Earlier theories noted that the human infant grows through play and fantasy in a ‘potential space’ and by using a transitional object aimed at mediating between fantasy and reality (Winnicott, 1991). The terms have been thereafter attributed to psychotherapeutic relations between the patient and the therapist. The ‘inanimate third’ is an object which resembles a shared unknown space for ‘mental digestion’ (Brown, 2012). There is an invasion of private space by using the ‘inanimate third’ in remote therapy as part of the rooms of both participants are visible. However, many other rooms and aspects of both the patient's and the therapist's privacy are unseen and remain for further reflections and fantasies. To do justice to the original meaning of the terms ‘transitional object’ and ‘potential space’ (Jones, 1992), it should be emphasized that the fantastic mindful operations embedded in these terms are aimed at emotional growth. Therefore, if the spoken does not equal the unspoken as much as the seen does not equal the unseen, the work with the ‘inanimate third’ leaves room for fantasy and thereby emotional growth.

Working with the ‘inanimate third’ may sound very concrete and without true connection to fantasy and imagination, hope and creativity or emotional growth. However, functional magnetic resonance imaging studies across ages show activity in brain regions related to emotional processing such as the amygdala and the entire corticolimbic system, resulting from audio or visual stimulations such as happy or sad pictures and sounds (Patel *et al*., 2012). Similarly, the child's doll, the widely accepted ‘transitional object’, is not just a concrete doll for the child. It is all the unspoken and unseen in the doll that triggers the child's development. Stimulation is concrete. The human mind is free and has individual horizons. This means that the distance does not have the power to stop fantasy from working in favour of human growth. On the contrary, if a therapeutic encounter is available, it works to stimulate the fantasy. This stimulation may result in more than just feeling better. It may result in enriching the patient's mind with ideas and concepts on emotional being, its origins and developmental pathways. By stimulating the intimate and individual world of fantasy through the ‘inanimate third’, the therapeutic encounter may elicit the sense of being alive emotionally and that the permission for hope is given.

The ‘inanimate third’ may be used to stimulate fantasy and emotional growth despite the difficulties during the pandemic. The reduction of the therapeutic encounter to a regular social interaction occurs when fantasy and hopes collapse (Ferber, 2002, 2006; Gut, 1989). This could happen in any type of therapy, not necessarily when working remotely. In fact, in remote therapy, the patient may feel that the sessions using the ‘inanimate third’ are a rescue from a very disturbed world. To quote one patient during a remote recent session: ‘I think you and me should keep our normal flow compared to the other things nowadays’.

*Vignette 6*: G, a child of a divorced couple, aged 10, diagnosed with autistic spectrum disorder, lived with his father in quarantine for three months and was not able to see his mother during this period. From the time of quarantine onset, the mother, the father and the child had separate remote therapy meetings each week. During quarantine, I discussed with the child his fantasy world of internet play games. After a week of returning to school, he was hyperactive in front of the camera, his face looked sad and his attention span was very limited. He turned the camera off a few times and explained that he wants to see me but does not want me to see him. Gradually he was convinced to turn on the camera. He said that he has something sad to say and mentioned that he wants his parents to get back together in the same house. He was encouraged by the ability to see his mother again. In the following days, he suffered from intrusive thoughts of his death and killing the father as announced by the school principle who was very troubled. I called the father, and he detailed a conversation with the child about the fact that the child considers that the father does not want to get back to the mother while the child is aware of the mother's wishes for a reunion. In the next session with the child, I appreciated his wish for this reunion and said that no child needs to be violent as in his internet games to attain the love of both his parents. I told him and his parents that every child of divorced parents wants the parents to be back together. The child said that his fighting with the intrusive thoughts is easier. He attributed this to my question if he tried to adapt to the difficult times by fantasizing on quarantine together with both parents. The following day, the father told me that he wishes to conduct a few sessions to process his preliminary wishes for the reunion with the mother before taking a serious step.

*Interpretation*: In this regard, my complex interactions with the child and his parents during quarantine stimulated the ultimate hope of the child to fix his world. This child has a personal interpretation of the therapeutic stimulation for emotional growth during quarantine. He thought about fixing his own world. He became more aware of his true individual needs during quarantine and separation from his mother, although he needed facilitation in realizing that his fantasies are aggressive while his needs focus on parental availability and love. He experienced this in a deviated way influenced by the violent scenes in his prolonged internet games during quarantine. This triggered intrusive thoughts and the entire family required professional aid to relate to the child's generating wish and hope concerning his parents.

### Between the ‘Uncanny’ and the ‘Inanimate’ Third: Are Transference and Countertransference Processes in Conditions of Electronic Interferences Possible?

Recent publications were concerned with the fate of central analytic processes such as transferential relations, therapeutic mirroring and containment when using remote therapy during fluent electronic communication and especially during bugged and interfered conditions (Sayers, 2021). Electronic devices provide, by definition, distorted auditory and visual stimuli compared with in vivo social encounters (Brahnam, 2017). The term ‘noise’ has been used to describe the electronic target for error corrections to reach a clear signal, which is nevertheless not identical to the original in vivo stimuli (e.g., signals are sharpened, colours heightened, noise adaptively suppressed, silence suppressed, and missing data and other aspects of the signals are synthesized and augmented, while above all communication quality has to be checked and rechecked, eliciting anxiety during communication breaks). Thus, the corrected signal after eliminating the noise has been suggested to restrict the analytic process. Accordingly, the term ‘noise’ has also been used to describe the unintentional verbal and non‐verbal messages during the analytic encounter (Brahnam, 2017). It has been claimed that these unintentional messages during the therapeutic encounter comprise the core of the analytic goal: unravelling the unconscious. This proposition raises the question of whether the electronic ‘noise’ and the error‐correcting aligned distortions of the signals for human sensory input interfere with the unconscious human natural ‘noise’, thus precluding the electronic devices from fulfilling their potential use in conducting an analytic encounter. It has been suggested that during virtual meetings nothing appears as it really is (Brahnam, 2017) and that work through ‘the uncanny third’, meaning the borderless powerful potential of the electronic device, may lead to an experience of an omnipotent symbiosis between the patient and the therapist. It has been further claimed that augmenting the inanimate device's representation in its user's mind creates an impression of living that is virtual and not real, thus in fact emphasizing the tension between life and death (Dettbarn, 2018). In addition, because the therapeutic couple is not necessarily mutually contained in a shared safe and facilitating environment, both members may unconsciously avoid the patient's regression. Given the limitations of remote therapy, we really cannot assume that what we are perceiving is what originated at the other end and phrases like ‘If I am understanding what you say correctly … ’ or ‘If I am hearing you right …’ are even more necessary.

To better understand whether the electronic device provides a distal proximity or interferes with the clinical and theoretical basis of the analytic encounter, the term ‘telepresence’ has been suggested. It suggests that the first order perception is the presence of the other in the electronic device and the second order perception is the other's presence in an extrapersonal space, a production of the human mind through processing embodiments of sensory inputs into a subjective perceptual extended experience (Haans & Ijsselsteijn, 2012). Accordingly, it is suggested here that this extended perceptual experience generates transference and countertransference processes within the virtual encounter of the analytic couple and creates the space for verbalized projections and their interpretation.

*Vignette 7*: Mr H and Mrs F, man and wife (respectively), married for 10 years, aged 30, parents of four children, were referred for treating the symptoms that looked to other medical staff as epileptic seizures experienced by Mrs F since her six miscarriages, which occurred prior to giving birth to four other children. During sessions, when her traumatic experiences were worked through, her symptoms occurred and they seemed to be better described as a dissociative state. When entering this dissociative state, her pupils were blocked and did not move, and she moved back and forth with her upper body, saying: ‘I am not with you, I will be back in a minute’. After a few minutes, she would smile and say: ‘Now I am back. I am with you’, without any more repetitive upper body movements and with free movement of her eye pupils and gaze. The diagnosis of post‐traumatic stress disorder with a dissociative symptom had been accepted by the couple.

In this particular session, Mr H said: ‘My wife is innocent, why is she suffering so much? I have been in our traumatic events as well and I put it behind. It passed. It is part of our history. Not part of our present life. We have four kids …’ I asked: ‘And are you saying you are happy and she is not?’ Mr H answered: ‘I am sorry for her. Without her symptoms everything should be OK’. I then asked Mrs F if she is happy with the children. She answered: ‘How could I be happy without him helping me with the kids?’ I then asked Mr H what blocks his availability for care and participation in the parental role? Mr H answered that he should stay undisturbed when working for the family's living expenses. I continued: ‘And what do you envision feeling if you change diapers for example?’ ‘I would have felt that I betray myself as a man’, Mr H answered. ‘So, we have here an issue with betrayal’, I said, and he answered: ‘Well, about infants and betrayal, this is an old story I should tell’. At this very moment the electronic communication broke up, I lost the couple and the couple lost me. I worried that Mrs F will experience her dissociative symptoms while there is only me, disconnected, to blame because I met her remotely and not face to face. I recalled Robert Frost's poem ‘The road not taken’ and felt guilty both for the road I had taken and for the one I had not taken. Following my countertransference feelings, I felt guilty for leading the session to this point and quite concerned what the story about betrayal was about. I tried to ring the couple to renew the communication and it did not work. With trembling fingers and emerging trepidation, I searched for their cell phone number just to ask if they were OK, but my anxious motor reactions blocked my ability to simply find their phone number in my cell phone list. I asked myself again and again if the husband is going to confess a love affair out of the session and should this be termed as acting out. At this point, after too many minutes of confusion, they rang and the videoconferencing device quality was good. I said: ‘Thanks God, you are back. I wondered what was going on with you two’. Then Mr H said: ‘I was about to tell you that there is a story in the family on my early infancy and I wish to tell you this. My parents moved to my grandparents’ house after I was born. My grandfather helped much with washing me, feeding and changing my diapers. His wife did not like my mother and interfered with my grandfather's help. My grandfather's help to my mother was generated by his affair and great love to her mother, ending in marrying a different woman who knew the story and therefore ignored me and my mother. My grandfather and my mother went along in caring for me better than my own father who was excluded’. I held my breath and said: ‘So, you feel that changing the diapers and helping your wife with your own children smell like a betrayal and you exclude yourself to a non‐paternal state of mind like your father? Perhaps you described your sorrow in detail earlier today as you excluded yourself from all happiness encompassed in father−child relations and the pride of being a father’. Mr H was silent and then Mrs F said as if she read my mind: ‘I knew you would be worried about me, but you are saving my marriage with these meetings online. I don't know how you are doing this, but this is my true experience. I am here. I did not disappear this time. Just your picture and your voice disappeared and came back as I always came back too’. I answered: ‘I felt guilty for betraying you two and leaving you to the mercy of the device. She answered: ‘Never worry about this. My husband is a good engineer’.

*Interpretation*: The session showed a process of de‐omnipotent experience by all parties as the capability and incapability were worked through: The capability of the mother not to collapse and to compare my disappearance from the session to her dissociative symptom, the capability of the father as an engineer and man of his word, and the capability of the therapist to make sense of the session despite the collapse of the device. In this sense, while the device collapsed, the human mind showed competence in living the past, the present and the future, not limited by the device. The countertransference feelings of guilt projected by the father's transference worked as a therapeutic engine for Mrs F. She analysed the emotional situation and spoke of gains in this session with a verbalized transferential projection of her dissociative condition on the interrupted connection with the therapist but without experiencing the symptom. Her ability to verbalize her symptom contributed to her increased conscious control of it. The family history of betrayal, which blocked the father's subjective paternal role and activity in childcare tied the couple together rather than separated, despite the fears of the therapist. The advantage of unpacking the unconscious and unspoken in this session was available as the therapist did not interfere with the emotional process of the patients before and after the collapse of the device. In fact, using Frost's words, the patients walked along the road they have taken, not one that could be shown by external agencies, such as the therapist. The transference−countertransference processes in this session, namely, the guilt feelings of the father and the ‘disappearances’ of the mother compared with the disappearance of the therapist and the session correspond to Freud's example of destruction and restoration in a child game mentioned as the game of ‘being gone’ (fortsein) or ‘disappearance and return’. The child, by holding a cot with a string, throwing a reel which disappeared in the cot, played with finding the reel again and again by the string (Freud, 1955). The restoration or finding and return process, in the old Freudian school, is the ultimate meaning of staying alive and further growing, as exhibited in therapies aimed at coping with the COVID‐19 pandemic stressors, to provide supplementary growth despite the life‐threatening and social compromising situation. Note that including electronic interferences in the interpretation to the patient brings the device back from its role as ‘uncanny’ to its true role as ‘inanimate’, showing the superiority of the human mind over the inanimate tools that it uses.

## CONCLUDING REMARKS: ON THE SOCIAL PARADOX

Two paradoxical concepts are widely discussed by Isaacs Russell (2021). Both are related to our concept of the ‘social paradox’. The first paradox is that everyone's pandemic is both shared and unique. The second paradox is between the experience of telepresence, where deep work can be done, and the breaking of telepresence, where the distance and device intrude, rendering the participants alone by technical interferences. The paradox of a relationship containing both distance in two environments and embodied closeness on a shared conscious and unconscious space also needs to be held in mind, eventually experienced and explored.

In our view, the paradox we identify, touches the axis of objectivity−intersubjectivity in psychoanalysis. For the past decades the therapeutic encounter is viewed as the subjective interaction between two subjective entities (Mitchell *et al*., 1999). The concept of uniqueness of the therapeutic interaction as determined by its mutual creation by both the therapist and the patient is borrowed from the intersubjective perspective. Aligned to the emphasis on subjectivity is the issue of symmetry between the therapist and the patient. No agreement has been reached as yet with regard to this symmetry. However, in the COVID‐19 pandemic both the therapist and the patient are subject to the same rules of social distancing and risks of being contaminated, although the psychological‐economical‐social conditions of the two may differ, even radically. In addition, in remote psychotherapy, an objective machine represents separation and unites the therapist and patient at the same time.

Thus, the issue of dancing between the subjective and objective worlds and between unity and separation is among the most prominent conflicts of carrying out psychotherapeutic processes during the pandemic. Do the needs, risks and social distancing unite the patient and the therapist, while they are physically separated? Can intersubjectivity occur from a distance? Can intersubjectivity and the mutual creation of the ‘analytic third’ (Benjamin, 2017) be based on objectivity of the ‘inanimate third’?

We suggest the term of ‘social paradox’ to describe the fact that an objective entity such as the digital media symbolizes both distance and intimacy, as well as separation and unity. Living within this paradox around the world may require trusting the logics of keeping a dialogue active. As the therapist and the patient are directly forced into a symmetrical interaction, it is the success of interaction cycles between them during the remote session which indicate the success of therapy. Both the therapist and the patient can start an interaction cycle and both can independently terminate it. Numerous interactions may be cycled through during the remote session.

The social paradox combines objectivity with subjectivity, unity and separation. The synchronicity of the therapeutic dialogue and the back‐and‐forth successful continuation of the dialogue between the patient and the therapist may determine the difference between regular social encounter and therapy.

Is the evolving mutual dialogue aimed to cure effective? Does it make us closer or more separated? To conclude, we think that containment of the social paradox by the therapeutic dialogue is possible as the existence of the dialogue eliminates elements of the paradox. As for working through a traumatic collective experience, we hope that both the therapist and the patient may feel cured as a result of their synchronous dialogue facilitated by the ‘inanimate third’. Emotional processes do occur from a distance and the mind is not dependent on location.

## Author note:

We have no conflicts of interest to disclose.

## NOTE

1 Confidentiality is ensured in the presentation of vignettes.
